# Identification of Specific Trafficking Defects of Naturally Occurring Variants of the Human ABCG2 Transporter

**DOI:** 10.3389/fcell.2021.615729

**Published:** 2021-02-09

**Authors:** Zsuzsa Bartos, László Homolya

**Affiliations:** Institute of Enzymology, Research Centre for Natural Sciences, Hungarian Academy of Sciences Centre of Excellence, Budapest, Hungary

**Keywords:** cellular routing, trafficking defect, glycosylation, kinetics, RUSH system, ABCG2 (BCRP)

## Abstract

Proper targeting of the urate and xenobiotic transporter ATP-binding transporter subfamily G member 2 (ABCG2) to the plasma membrane (PM) is essential for its normal function. The naturally occurring Q141K and M71V polymorphisms in ABCG2, associated with gout and hyperuricemia, affect the cellular routing of the transporter, rather than its transport function. The cellular localization of ABCG2 variants was formerly studied by immunolabeling, which provides information only on the steady-state distribution of the protein, leaving the dynamics of its cellular routing unexplored. In the present study, we assessed in detail the trafficking of the wild-type, M71V-, and Q141K-ABCG2 variants from the endoplasmic reticulum (ER) to the cell surface using a dynamic approach, the so-called Retention Using Selective Hooks (RUSH) system. This method also allowed us to study the kinetics of glycosylation of these variants. We found that the fraction of Q141K- and M71V-ABCG2 that passes the ER quality control system is only partially targeted to the PM; a subfraction is immobile and retained in the ER. Surprisingly, the transit of these variants through the Golgi apparatus (either the appearance or the exit) was unaffected; however, their PM delivery beyond the Golgi was delayed. In addition to identifying the specific defects in the trafficking of these ABCG2 variants, our study provides a novel experimental tool for studying the effect of drugs that potentially promote the cell surface delivery of mutant or polymorphic ABCG2 variants with impaired trafficking.

## Introduction

The human ATP-binding transporter subfamily G member 2 (ABCG2) [breast cancer resistance protein (BCRP)], a member of the ATP-binding cassette (ABC) transporter superfamily, mediates the transport of numerous metabolites and xenobiotics at various physiological barriers. Among others, it facilitates urate excretion in the kidney and the gastrointestinal tract (Borst and Elferink, 2002; van Herwaarden and Schinkel, 2006; Giacomini et al., 2010). This transporter with its broad substrate recognition is also implicated in multidrug resistance of cancer cells. ABCG2 is localized in the plasma membrane (PM) and resides predominantly in the apical domain when expressed in polarized cells. Since the primary task of this transporter is the extrusion of substances from the cell, correct trafficking to the proper membrane compartment is crucial for its function.

Following biosynthesis on the surface of the endoplasmic reticulum (ER), ABC proteins, like other membrane proteins, get folded and inserted into the ER membrane. Several PM-resident ABC proteins then get glycosylated. This process is initiated in the ER and completed in the Golgi apparatus. ABCG2 has been reported to be glycosylated on asparagine 596 (Diop and Hrycyna, 2005). Some early studies reported that mutations at this residue do not affect the expression and trafficking of ABCG2 (Diop and Hrycyna, 2005; Mohrmann et al., 2005); however, a more sensitive assay subsequently demonstrated a partial proteasomal degradation of ABCG2 when N-linked glycosylation was pharmacologically disrupted or N596 was mutated (Nakagawa et al., 2009). After maturation, PM-resident ABC proteins are directed from the Golgi apparatus to the cell surface. Certain ABC proteins, exemplified by ABCC2/MRP2, are delivered directly to the PM (Zeigerer et al., 2012), whereas others, such as ABCB11/BSEP, traffic to an endosomal pool first and subsequently get delivered to the cell surface (Kipp et al., 2001; Wakabayashi et al., 2005). ABCB1/MDR1 has been reported to take both direct and endosomal routes (Sai et al., 1999; Kipp and Arias, 2000; De Rosa et al., 2004; Wakabayashi et al., 2005; Fu et al., 2007). Moreover, under certain conditions, non-glycosylated ABCC7/cystic fibrosis transmembrane conductance regulator (CFTR) traffics directly from the ER to the PM, bypassing the Golgi apparatus, via a Golgi reassembly stacking protein (GRASP)-dependent pathway (Gee et al., 2011). The particular trafficking routes, taken by ABCG2 on its journey from the ER to the cell surface, remain to be elucidated.

Some mutations or polymorphisms in the genes encoding for ABC transporters do not affect the proteins’ transport activity, but rather their cellular trafficking. The most widely known of these is the ΔF508 mutation in *CFTR*, which is the most frequent genetic determinant of the hereditary disease, cystic fibrosis (Lukacs et al., 1993). Several other mutations in various ABC transporters have been reported to cause trafficking impairment. When the function is more or less preserved, these defects can at least partially be restored by small molecules called pharmacological chaperones or correctors. Corr-4a and Lumacaftor (VX-809) have been specifically developed to improve the trafficking of CFTR mutants (Ren et al., 2013), whereas a histone deacetylase inhibitor, 4-phenylbutyric acid (4-PBA), has proven to be effective in ameliorating cell surface delivery of numerous ABC transporter variants with impaired trafficking. These include mutants of CFTR, ABCB4/MDR3, ABCB11/BSEP, ABCC6/MRP6, as well as ABCG2 (Rubenstein et al., 1997; Hayashi and Sugiyama, 2007; Woodward et al., 2013; Gordo-Gilart et al., 2016; Pomozi et al., 2017).

Numerous mutations or polymorphic variations in ABCG2 have been reported (Heyes et al., 2018). Of these, several result in diminished trafficking of the transporter (recently reviewed in Mozner et al., 2019). The most well-studied polymorphism in ABCG2 is a glutamine-to-lysine substitution at residue 141 (Q141K) (Imai et al., 2002). The allele frequency of this polymorphism is the highest in the Asian population (17%), but it is also close to 10% in the Caucasian population. A strong link between the Q141K-ABCG2 and the development of gout has been established (Woodward et al., 2013). Recently, we have identified and characterized a novel polymorphic variant (M71V) of ABCG2 (Zambo et al., 2018). In that previous study, a group of healthy volunteers and patients with clinically verified gout or hyperuricemia was screened for low ABCG2 expression levels. In individuals with markedly reduced ABCG2 expression, the *ABCG2* gene was sequenced, leading to the identification of the M71V variant. The frequency of this variant in the general cohort was about 1%. The relatively small group size of patients did not allow us to draw any conclusions regarding whether M71V is more frequent in individuals with gout or hyperuricemia. A systematic screening involving more patients is still required to clarify this issue.

A detailed characterization of this novel ABCG2 variant revealed that M71V-ABCG2 is similar to the well-characterized Q141K variant in several aspects (Zambo et al., 2018). Both Q141K and M71V exhibit a somewhat reduced transport activity, but predominantly, they have major folding and trafficking defects. Their expression levels are reduced as a consequence of increased degradation. Interestingly, inhibition of either proteasomal or lysosomal degradation leads to an increased expression of Q141K-ABCG2, demonstrating the involvement of both of these mechanisms in the degradation of this variant (Nakagawa et al., 2009; Basseville et al., 2012). The accumulation of Q141K in perinuclear compartments, identified as aggresomes, has also been demonstrated (Basseville et al., 2012). The expression level of M71V is also increased upon proteasome inhibition (Zambo et al., 2020). As regards cellular distribution, the majority of both Q141K and M71V are localized intracellularly, but a small fraction of both variants can reach the PM (Zambo et al., 2018, 2020). Although substantial information on the Q141K variant has been accumulated, it is still not fully understood how this or the M71V polymorphism actually affects the cellular trafficking of ABCG2.

For studying cellular trafficking, the most commonly used method is immunostaining combined with microscopy. However, this approach gives information only on the steady-state distribution of the cellular component of interest. Direct labeling of proteins with genetically encoded fluorescent tags, such as fluorescent proteins, reinvigorated the trafficking studies of proteins. Studying the dynamics of cellular routing also requires a sort of synchronization, which is analogous to the “pulse” in a pulse-chase experiment in biochemistry. Synchronization can be performed by photoactivation, photoconversion, thermal block, or using trapping tags, such as conditional aggregation domains or thermosensitive viral glycoprotein (VSVGts). Recently, a novel method, named the Retention Using Selective Hooks (RUSH) system, also relying on the use of trapping tags, has been reported (Boncompain and Perez, 2012). This synchronization method is based on the interaction of streptavidin with streptavidin-binding peptide (SBP) and biotin.

In the present study, we have investigated the cellular routing of the wild type (wt) ABCG2, as well as the Q141K and M71V polymorphic variants, focusing on their delivery from the site of synthesis (ER) to the business end, i.e., the PM. To explore the trafficking kinetics of the ABCG2 variants, we employed the RUSH system. This method allowed us to dissect the various stages of cellular routing, as well as to identify the particular trafficking events that are impaired by the Q141K and M71V variations. Besides deciphering the specific routing defects of the ABCG2 variants, our study also provides a novel experimental tool for studying ABCG2 trafficking and screening drugs that may facilitate the cell surface delivery of mutant/polymorphic variants with trafficking defects.

## Materials and Methods

### Materials

Biotin (Sigma-Aldrich, cat. B4501) and 4-PBA (Sigma-Aldrich, cat. P21005) were dissolved in distilled water and used at 100 μM and 1 mM final concentrations, respectively. MG132 (Sigma-Aldrich, cat. M7449), Bafilomycin A1 (BAF) (Sigma-Aldrich, cat. B1793), and Ko143 (Sigma-Aldrich, cat. K2144) were dissolved in dimethyl sulfoxide (DMSO) (Sigma-Aldrich, cat. 276855) and applied at 2 μM, 10 nM, and 1 μM final concentrations, respectively. For solvent controls, distilled water and DMSO were used accordingly.

### DNA Constructs

Sequences of the M71V and Q141K ABCG2 variants were recloned from other vectors previously generated in laboratory (Zambo et al., 2018 and Zs. Bartos unpublished) into the pcDNA_3.1-EGFP-ABCG2 plasmid (Orban et al., 2008). The various RUSH-ABCG2 expression vectors (wt, M71V, Q141K) were generated from the ER hook-containing Str-Ii_SBP-EGFP-Golgin84 plasmid (Addgene # 65303), in which the EGFP-Golgin84 sequence was replaced with various EGFP-ABCG2 sequences by cloning them with the following primers: atggacgagctgtacaagggactcagatctcgag (forward) and ggctgattatgatcagttatcagttatctagatccggtggatc (reverse) using the Gibson assembly method (New England BioLabs, NEBuilder HiFi DNA Assembly Master Mix, cat. E2621). The sequences of all plasmid constructs were confirmed by Sanger sequencing (Microsynth AG). In certain experiments, a plasmid containing the sequence of untagged wt ABCG2 (pcDNA_3.2-ABCG2) was also used (Orban et al., 2008).

### Cell Culture and Transfection

HeLa cells were maintained in Dulbecco’s Modified Eagle’s medium (D-MEM)/high glucose/GlutaMAX (Gibco, cat. 10569010), completed with 10% FBS (Gibco, cat. 1640071) and 1% penicillin–streptomycin (Gibco, cat. 15070063) at 37°C (5% CO_2_). Transient transfection of HeLa cells was carried out with Lipofectamine 2000 (Invitrogen, cat. 11668019) in Opti-MEM (Gibco, cat. 31985070) according to the manufacturer’s protocol. Cells for qPCR and Western blotting were grown on 12-well plates (Greiner, cat. M8687), whereas those used for microscopy were seeded onto ibi-Treat μ-Slide eight well chambers (Ibidi, cat. 80826). HeLa cell line stably expressing wt ABCG2 has been previously generated in our laboratory (Zambo et al., 2020).

### RNA Extraction and Real-Time PCR

mRNA samples from cells expressing RUSH-ABCG2 were isolated and purified 24 h after transfection using a PureLink MiniKit (Thermo Fisher Scientific, cat. 12183018A) according to the manufacturer’s instructions. After cDNA conversion (Thermo Fisher Scientific, High-capacity cDNA reverse transcription kit, cat. 4368814), PCR reactions were run on a StepOnePlus^TM^ platform (Thermo Fisher Scientific) using qPCR probes for ABCG2 (Thermo Fisher Scientific, cat. 00184979) and RPLP0 (Thermo Fisher Scientific, cat. 99999902). ABCG2 mRNA levels were normalized first to RPLP0 and then to ABCG2-wt mRNA levels using the ΔΔCt method.

### Western Blotting and Glycosidase Digestions

Protein from the untreated cells (0 h) as well as from those subjected to 100 μM biotin for the indicated times was extracted by the addition of TE buffer (0.1 M TRIS-PO_4_, 4% SDS, 4 mM Na-EDTA, 40% glycerol, 0.04% bromophenol blue, and 0.04% β-mercaptoethanol; materials from Sigma-Aldrich), and then the samples were sonicated. In certain experiments, when indicated, the cells were pre-treated with 1 mM 4-PBA, 2 μm MG132, or 10 nM BAF overnight prior to biotin addition. Equal amounts of the protein samples were loaded onto 7.5% polyacrylamide gels. For glycosidase treatments, cell lysates containing 20 μg of protein were subjected to 20 U Endoglycosidase H (Endo H) (Sigma, cat.11088726001) or 20 U PNGase F (Roche, cat.11365185001) according to the manufacturer’s protocols. Blots were probed with anti-ABCG2 (Bxp-21, Abcam, cat. ab3380) or anti-β-actin (Sigma, cat. A1978) primary antibodies, and subsequently developed with HRP-conjugated goat anti-mouse IgG (H + L) secondary antibody (Abcam, cat. ab97023). Detection was performed by luminography using Clarity Western ECL Substrate (Bio-Rad, cat. 1705060). For quantification, densitometry was carried out by the ImageJ software.

### Immunostaining of Fixed Cells

HeLa cells, previously seeded onto an eight-well chamber at 2 × 10^4^ cells/well density and grown for 24 h, were transfected with the RUSH-ABCG2 variants as described above. Twenty-four hours after transfection, the cells were subjected to 100 μM biotin for the indicated times (or remained untreated–0 h). Following this, the samples were gently washed with PBS and then fixed with 4% PFA for 10 min at room temperature. After several washing steps, the cells were blocked for 1 h at room temperature in Dulbecco’s modified phosphate buffer saline (DPBS) containing 2% bovine serum albumin, 1% fish gelatine, 0.1% Triton X-100, and 5% goat serum (blocking buffer). Next, the samples were incubated with anti-Giantin primary antibody (1:1000 in blocking buffer, BioLegend, cat. 924302) at 4°C overnight. After being washed with PBS, the cells were incubated with Alexa Fluor-594 conjugated anti-rabbit secondary antibody (1:250 in blocking buffer, Thermo Fisher Scientific, cat. A11012) for 1 h at room temperature.

### Labeling ER and Cell Surface Expression of ABCG2 in Live Cells

For ER labeling, 24 h after transfection and following biotin treatment (when applicable), the cells expressing the RUSH-ABCG2 variants were subjected to ER-Tracker Red (1:1000 in Hanks’ Balanced Salt solution, Thermo Fisher Scientific, cat. E34251) for 30 min at 37°C. Next, the samples were gently washed with PBS, fixed with 4% PFA for 10 min at room temperature, and washed again three times with PBS. For a better understanding of the labeling procedure, see Supplementary Figure 1A. To label ABCG2 on the cell surface, 24 h after transfection, the cells expressing the RUSH-ABCG2 variants were co-incubated with Alexa Fluor 647 conjugated 5D3 antibody (1 μg/ml, Novus Biological and Biotech, cat. FAB995R), 1 μM Ko143 (Sigma-Aldrich, cat. K2144), and 100 μM biotin in D-MEM for various lengths of time ranging 1–8 h. For time zero, biotin was omitted from the medium (Supplementary Figure 1C). Subsequently, the samples were gently washed with PBS, fixed with 1% PFA for 5 min at room temperature and washed again three times with PBS.

### Confocal Microscopy Imaging and Colocalization Analysis

Cells were imaged by a Zeiss LSM 710 laser scanning fluorescence confocal microscope using a Plan-Apochromat 40 × (N.A. = 1.4) oil immersion objective. For each condition, six images were acquired at each time point. Each field of view contained 15–20 transfected cells identified by the green fluorescent protein (GFP) fluorescence. The green fluorescence of GFP was acquired between 500 and 540 nm at 488 nm excitation. When applicable, the far-red fluorescence over 640 nm was acquired sequentially with the green signal at 633 nm excitation. The images were analyzed by the ZEN 2012 software. Colocalization was expressed by the colocalization coefficient (*CC*) as follows:

$$C\,C=\frac{\sum N_{G,c\,o\,l\,o\,c\,a\,l\,i\,z\,e\,d}}{\sum N_{G,t\,o\,t\,a\,l}}$$

where *N*_*G*,__colocalized_ denotes the number of colocalized (green and red) pixels and *N*_*G,total*_ refers to the total number of pixels in the green channel. It is important to note that unlike Pearson’s or Manders’ coefficients, *CC* avoids using pixel intensities but is based on numbers of pixels, thus demonstrating overlapping. For determining *CC* values, the automatic Costes threshold method was used in the ZEN software. In some experiments, when indicated, not *CC* but the integrated far-red fluorescence (single channel) was determined.

### Kinetic and Statistical Analyses

After determining the kinetics of the appearance of the ABCG2 variants in various compartments, the time courses of the *CC* were fitted with different equations using the least square method. For release from the ER, a single exponential decay was applied as follows:

$$C\,C_{E\,R}=C\,C_{0}\cdot e^{-k_{o\,u\,t}\cdot t}+C\,C_{\infty }$$

where *t* and *CC*_*ER*_ are the independent and dependent variable, respectively; *CC*_0_, *CC*_∞_, and *k*_*out*_ are free parameters. *CC*_0_ gives the initial value (*t* = 0), whereas *CC*_∞_ indicates the limit of the function (*t* = ∞), and *k*_*out*_ is the time constant. For the transit through the Golgi apparatus, two exponential decay functions embedded in one another was employed:

$$C\,C_{G\,o\,l\,g\,i}=\left(C\,C_{0}-C\,C_{\infty }\right)\cdot e^{-k_{i\,n}\cdot t}+\left(1-e^{-k_{i\,n}\cdot t}\right)\cdot e^{-k_{o\,u\,t}\cdot t}+C\,C_{\infty }$$

The designations are similar to that above, with the difference that there are four free parameters here: *CC*_0_, *CC*_∞_, *k*_*in*_, and *k*_*out*_. The two time constants (*k*_*in*_ and *k*_*out*_) refer to the inward and outward transport, respectively. Delivery to the PM was described with a sigmoidal function:

$$C\,C_{P\,M}=\frac{A}{1+e^{-k_{i\,n}\cdot \left(t-\Delta \,t\right)}}+B$$

where *A*, *B*, and Δ*t* are free parameters. The initial value and the convergence can be calculated from these parameters as follows:

$$C\,C_{0}=\frac{A}{1+e^{k_{i\,n}\cdot \Delta \,t}}+B$$

$$C\,C_{\infty }=A+B$$

For studying surface delivery, not only *CC*_*PM*_ but also the integrated far-red fluorescence was fitted. For curve fitting, Microsoft Excel Solver, a part of the Analysis ToolPak Add-in, was used applying the GRG Non-linear engine, which is applicable for solving smooth non-linear problems. The variables were altered until the sum of squared differences between the measured and fitted points reached the minimum. Fitting was performed with three independent experiments involving 100–120 cells each.

For statistical analyses, Student’s *t* test was used. Differences were considered significant, when *p* < 0.05.

## Results and Discussion

### Generation and Characterization of ABCG2-RUSH Expression Vectors

Certain polymorphisms in ABCG2, such as the M71V and Q141K substitutions, impair cellular trafficking of the transporter, but leave its transport function more or less unaffected (Zambo et al., 2018). Our aim was studying how these two polymorphisms affect the most crucial part of cellular trafficking of a PM resident protein: the delivery from the ER to the cell surface. To analyze the trafficking of the ABCG2 variants in detail, we employed the dynamic RUSH system (Boncompain and Perez, 2012). In this synchronization method, the so-called hook protein, resident of a specific cellular compartment (donor compartment), is tagged with streptavidin, whereas the protein of interest (reporter) is tagged with SBP. Besides, the reporter is fused to a fluorescent protein, e.g., GFP. Because of the strong interaction between streptavidin and SBP, the reporter is retained in the donor compartment by the hook when they are co-expressed. In turn, the protein of interest can be released by the addition of biotin, which has higher affinity to streptavidin than SBP has. This way, the protein of interest can be tracked from the donor compartment to its final destination.

**FIGURE 1:**
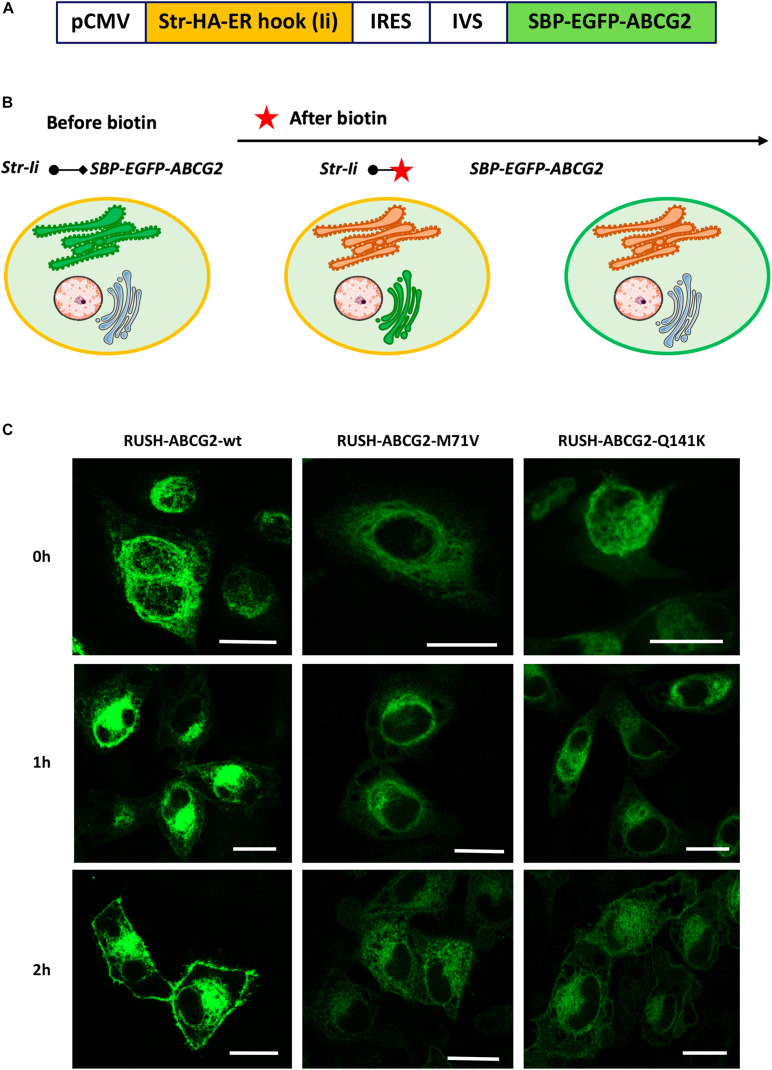
Characterization of the RUSH vectors containing sequences for ABCG2 variants. **(A)** Scheme of the expression vector encoding for the ER-hook (invariant chain, Ii) fused with a streptavidin (Str) tag, as well as for the ABCG2 N-terminally tagged with streptavidin-binding peptide (SBP) and GFP. **(B)** Principle of the RUSH system: in the absence of 100 μM biotin, the reporter is retained in the ER by the Str–SBP interaction. Upon the addition of biotin (marked with a red star), the SBP-EGFP-ABCG2 reporter is released from the ER, passes through the Golgi apparatus, and ultimately traffics to the plasma membrane. Panel **(B)** was prepared using image vectors from Servier Medical Art (www.servier.com). **(C)** Representative images of HeLa cells transiently expressing various RUSH-ABCG2 variants (wild type–wt, M71V, and Q141K) in the absence of biotin (0 h), as well as 1 and 2 h after biotin was added. The green fluorescence of GFP was acquired by confocal microscopy. Scale bars represent 20 μm.

For our studies, we used an ER hook, the invariant chain (Ii) of major histocompatibility complex (MHC) class II, and three variants of the ABCG2 as reporters: the wt, M71V-, or Q141K-ABCG2 (Figure 1A). In the bicistronic plasmid construct, the sequences of the hook and the reporter are under the control of cytomegalovirus (CMV) promoter and separated by an internal ribosome entry site (IRES) as well as a synthetic intron [intervening sequence (IVS)]. It is important to note that in our construct, the hook precedes the ABGG2 reporter. This consideration is based on the fact that the first sequence is translated more efficiently than the one following IRES (Mizuguchi et al., 2000); thus, this arrangement can minimize the leak from the donor compartment. The scheme in Figure 1B depicts the process to be studied: the synchronized release of ABCG2 from the ER and its delivery to the PM.

To validate the applicability of our RUSH vectors, we transiently transfected HeLa cells with the constructs containing the sequences of the ABCG2 variants and monitored their expression and cellular localization by means of the GFP fluorescence using confocal microscopy. As documented in Figure 1C, the SBP-tagged ABCG2 variants are efficiently retained in the ER without biotin (0 h). Upon addition of 100 μM biotin, the wt ABCG2 rapidly trafficked to the cell surface. One hour after treatment, it was predominantly localized perinuclearly (presumably in the Golgi compartment), and after an additional hour, it was mainly observed at the cell periphery (presumably in the PM). Two hours after biotin addition, about 40% of the total wt ABCG2 was localized to the cell periphery, whereas roughly 30% was perinuclear. In contrast, the M71V and Q141K variants were predominantly found intracellularly 2 h after biotin addition (about 25% of total perinuclearly), and only minor fractions of those were observed at the cell periphery (∼10 and ∼20%, respectively). A more detailed, quantitative analysis on the cellular distribution of ABCG2 variants is performed in subsequent experiments. These observations indicate impaired trafficking of the polymorphic variants as compared to that of the wt ABCG2.

**FIGURE 2:**
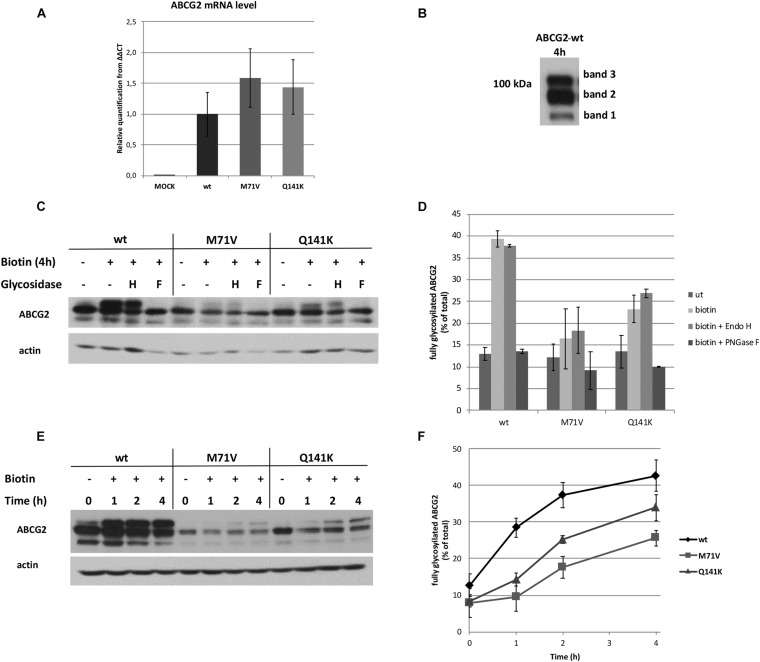
Expression and glycosylation of the ABCG2 variants before and after ER release. **(A)** The mRNA expression of RUSH-ABCG2 variants 24 h after transfection of HeLa cells. The expression levels were normalized to a reference gene (ribosomal protein P0) and to the level of wild-type ABCG2. Values depict mean ± SEM (*n* = 3). **(B)** Representative Western blot of RUSH-ABCG2-wt expressed in HeLa cells 4 h after biotin addition. **(C)** Deglycosylation of the ABCG2 variants by Endo H (H) and PNGase F (F) glycosidases in the absence of biotin and 4 h after biotin was added. **(D)** The fraction of the upper bands with or without glycosidase treatment. The intensities were determined by densitometry and expressed as % of total ± SEM (*n* = 2). **(E)** Western blot analysis of the ABCG2 variants retained in the ER (0 h) or 1, 2, and 4 h after synchronized release from the ER. **(F)** The kinetics of protein maturations of the ABCG2 variants. The fraction of the upper bands were determined by densitometry and expressed as % of total ± SEM (*n* = 3).

It is noteworthy that the polymorphic variants exhibited lower fluorescence intensities (Figure 1C), suggesting lower expression levels. Therefore, we next assessed the mRNA and protein expression levels by real-time PCR and Western blotting, respectively. As expected, no significant difference in the mRNA expression of the ABCG2 variants was seen (Figure 2A). This finding demonstrates that neither the transcription of the plasmids, nor the mRNA stability is affected. In contrast, marked differences in the protein expression levels were found (Figures 2C,E). The polymorphic variants exhibited considerably lower protein levels as compared to the wt. This observation is in line with previous findings demonstrating lower protein expression levels in cell lines transiently or stably expressing these ABCG2 variants (Zambo et al., 2018, 2020). Also, the ABCG2 protein levels were lower in the red blood cell membrane of individuals carrying these polymorphisms (Zambo et al., 2018). As mentioned earlier, the M71V and Q141K variants are subjected to proteasomal degradation. Co-expression with an ER hook could theoretically prevent these variants from entering the degradative pathway, but our recent finding suggests that the studied polymorphic variants get at least partially degraded before reaching the hook protein in the ER.

### Assessment of Maturation of the ABCG2 Variants

Western blot analysis revealed that ABCG2 appeared in three individual bands following biotin treatment (Figure 2B). It seems plausible that the lower band (band 1) on the Western blot corresponds to the non-glycosylated form of the transporter, whereas the middle (band 2) and upper (band 3) bands are the core and fully glycosylated forms, respectively. To explore this assumption, the ABCG2-containing samples were subjected to Endo H or PNGase F glycosidase treatment. No change in the apparent molecular weights was observed upon Endo H digestion, whereas only the two lower bands remained visible after the PNGase F treatment (Figure 2C). Endo H cleaves between N-acetylglucosamine residues of high mannose and some hybrid types of N-linked glycans, while PNGase F removes the entire carbohydrate tree from all types of N-linked glycoproteins. Therefore, our observations suggest that band 3 is indeed the fully glycosylated form of ABCG2. Western analysis of the wt ABCG2 alone revealed a small, but definite downward shift in the apparent molecular weight of band 2 upon PNGase F treatment (Supplementary Figure 2A), which is not visible in Western blots with longer exposures. It implies that band 2 contains both the core and non-glycosylated forms. Nevertheless, the identity of band 1 remains elusive. In previous studies, when ABCG2 was subjected to Western blot analysis, usually only two bands were reported. However, in some studies, a faint band can be observed below the two dominant bands, when cropping of the Western blot allows seeing this (Nakagawa et al., 2008; Basseville et al., 2012). Some other reports also demonstrated that treatment with PNGase F or tunicamycin resulted in three bands in Western blots assessing ABCG2 (Ozvegy et al., 2001; Hou et al., 2013). It can be assumed that this third, lower band is identical to our band 1, and likely is a degradation product. In subsequent quantitative assessments, the level of glycosylation of the ABCG2 variants was determined by the ratio of the upper band intensity over the total intensities of all three bands. The changes in the fraction of the fully glycosylated forms upon glycosidase treatments are shown in Figure 2D.

Next, we examined the kinetics of glycosylation of the different ABCG2 variants following release from the ER. At time zero (in the absence of biotin), the two lower bands were prevalent, although a faint upper band was also observed, especially for the wt (Figure 2E). It is most likely due to the minor basal leak from the donor compartment. Upon the addition of biotin, band 3 got gradually augmented and became comparable with band 2 in the case of the wt and the Q141K variant, whereas the maturation of the M71V variant was slower and less pronounced (see Supplementary Figure 2B). Interestingly, the total expression of wt ABCG2 exhibited a transient increase upon biotin addition, which eventually returned to the baseline (Supplementary Figure 2C). It is plausible that this transient alteration in the total protein level is a consequence of perturbation of the steady state of protein translation, folding, processing, and degradation. This transient was not observed with the polymorphic variants (Supplementary Figure 2C), implying that one or more of the cellular processes listed above differ from that seen with wt ABCG2, resulting in a different steady state without biotin.

Quantitative analysis of several Western blots similar to that shown in Figure 2E revealed delayed and confined glycosylation kinetics for the polymorphic variants as compared to that observed with the wt (Figure 2F). A detailed analysis of the kinetics of all three bands is shown in Supplementary Figure 2B. Four hours after biotin addition, the fully glycosylated fraction of the wt ABCG2 reached 42.7 ± 4.3%, whereas the share of band 2 per total declined from 63.6 ± 9.5 to 47.2 ± 3.1%. Similarly, the fraction of band 3 of the Q141K variant increased to 33.9 ± 3.5%, while the share of band 2 declined to 53.6 ± 3.8% after 4 h. The rise of the fully glycosylated form (band 3) was less pronounced for the M71V variant, reaching only 25.7 ± 2.1%, whereas band 2 declined to 62.8 ± 4.0% in this case. Since the glycosylation of membrane proteins is initiated in the ER and finalized in the Golgi apparatus, the observed glycosylation kinetics suggests that a substantial fraction of the ABCG2 variants (even that of the Q141K and M71V) is released from the ER and reaches the Golgi apparatus. In addition, our results also revealed a somewhat deferred and restricted maturation of the polymorphic ABCG2 variants.

### Dissection of ABCG2 Trafficking From the ER to the Cell Surface

To analyze the movement of ABCG2 from the ER to the cell surface, we expressed RUSH-ABCG2-wt in HeLa cells and labeled them with various cellular markers in the absence or presence of biotin. To monitor the release of ABCG2 from the ER, ER-Tracker Red was used (Supplementary Figure 1A), whereas to follow its transfer through the Golgi apparatus, immunostaining with anti-Giantin was performed (Supplementary Figure 1B). To assess the PM delivery of ABCG2, we employed Alexa Flour 647 conjugated 5D3 antibody, which recognizes extracellular epitopes of ABCG2. Since 5D3 labeling is conformation sensitive (Özvegy-Laczka et al., 2005), the cells were incubated with the antibody in the presence of 1 μM Ko143, a specific inhibitor of ABCG2 (Supplementary Figure 1C). Since labeling was preserved even after internalization (see Supplementary Figures 3A,B), all the ABCG2 that once reached the cell surface was detected, allowing us to monitor cell surface delivery by itself.

**FIGURE 3:**
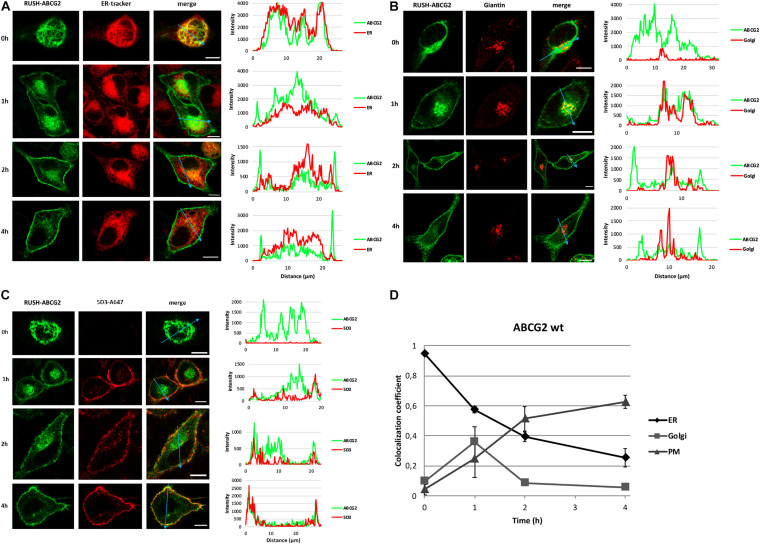
Tracking down ABCG2 from the ER to the cell surface. HeLa cells transfected with RUSH-ABCG2-wt were labeled with various cellular markers in the absence (0 h) and after the addition of biotin (1–4 h) as indicated. Representative confocal images depict the cellular localization of ABCG2 (green) and the marker (red). Intensity profiles on the right indicate the green and red fluorescence signals along a representative line (marked with a blue arrow) through a single cell. **(A)** The endoplasmic reticulum was labeled with ER-Tracker Red. **(B)** The Golgi apparatus was immunostained with anti-Giantin antibody. **(C)** Alexa Fluor 647 conjugated ABCG2-specific antibody (5D3) was used to detect ABCG2 on the cell surface. The detailed labeling protocols are shown in Supplementary Figure 1. Scale bars represent 10 μm. **(D)** Quantitative evaluation of the trafficking of ABCG2 from the ER to the PM. The fractions of ABCG2 present in various cellular compartments (ER, Golgi, and PM) were determined at different time points by the colocalization coefficient (the number of colocalized pixels over the number of all green pixels) in a series of confocal images similar to those shown in Panels **(A–C)**. The colocalization coefficients are plotted versus time (mean ± SEM of three independent experiments involving ∼120 cells each).

As demonstrated in Figure 3A, the GFP signal initially colocalized with the fluorescence of ER-Tracker Red, but got eventually separated, demonstrating the gradual release of wt ABCG2 from the ER. At time zero, the GFP-tagged ABCG2 did not colocalize with the Golgi marker, but exhibited an overlap with that 1 h after biotin addition (Figure 3B). Later, the two signals diverged again, indicating the transient appearance of wt ABCG2 in the Golgi apparatus. No wt ABCG2 was observed in the PM at time zero, but a small fraction of that appeared there 1 h after release from the ER, and the majority of the transporter reached the cell surface within 4 h (Figure 3C). To evaluate the trafficking events quantitatively, we performed a colocalization analysis using a series of images similar to those shown in Figures 3A–C. Instead of using the usual Pearson’s correlation coefficient, we employed the *CC*, as this measure expresses what fraction of ABCG2 is localized to the particular compartment designated by the specific cellular marker, independently of how many non-transfected cells are present in the studied field of view. This analysis revealed a rapid release of ABCG2 from the ER, its transient appearance in the Golgi apparatus, and its continuous delivery to the cell surface (Figure 3D). Over 60% of the wt ABCG2 reached the PM within 4 h. When the cells were incubated with biotin and 5D3 for a longer period of time, only a slight further increase was observed, indicating that PM delivery was essentially completed within 4 h (Supplementary Figure 3A). To verify that the 5D3-containing solution is not depleted of the antibody and is still able to detect ABCG2 on the cell surface even after longer incubation times, the supernatant was removed after 6 h and transferred to HeLa cells previously transfected with GFP-ABCG2 harbored in a regular (non-RUSH) plasmid. As demonstrated in Supplementary Figure 3B, the used supernatant is still applicable. It should, however, be noted that longer incubation times resulted in the internalization of ABCG2 as demonstrated in Supplementary Figure 3C. Internalization occurs normally at a certain speed, but 5D3 is known to accelerate endocytosis of ABCG2 (Studzian et al., 2015). In long-term experiments, *de novo* synthesis also feeds the pool of the studied protein; therefore, the system eventually converges to the steady-state arrangement. To avoid that, protein synthesis can be blocked by cyclohexamide. However, in our hand, cyclohexamide was found to exert no substantial effect in the short-term experiments, while it was detrimental to the cells upon extended treatment. Considering all these aspects, we focused on the first 4 h of ABCG2 trafficking in further experiments.

### Trafficking of the Polymorphic ABCG2 Variants

**FIGURE 4:**
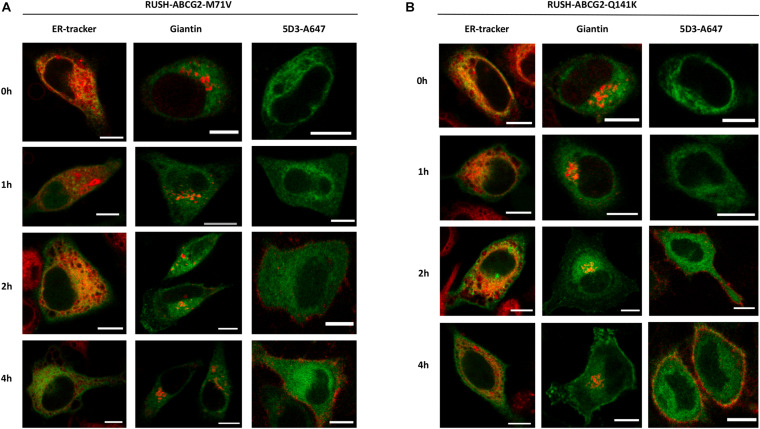
Trafficking of the ABCG2 variants from the ER to the cell surface. HeLa cells expressing the M71V-ABCG2 **(A)** or Q141K-ABCG2 **(B)** variants were labeled with various cellular markers in the absence (0 h) and after the addition of biotin (1–4 h). Representative confocal images show the cellular localization of the ABCG2 variant (green) and the marker (red). For more details, see the legend of Figure 3. Scale bars represent 10 μm.

Similar to that shown with the wt ABCG2 in Figure 3, the various stages of ER to PM trafficking of the M71V and Q141K variants were also studied. Interestingly, after the addition of biotin, a large portion of the polymorphic variants was retained in the ER, as indicated by their colocalization with the ER tracker (Figures 4A,B, left columns). A smaller fraction, however, reached the Golgi apparatus, and eventually the PM (middle and right columns, respectively). The PM localization of these variants is clearly indicated by the intensity profile analysis of GFP and 5D3 labeling shown in Supplementary Figure 4.

**FIGURE 5:**
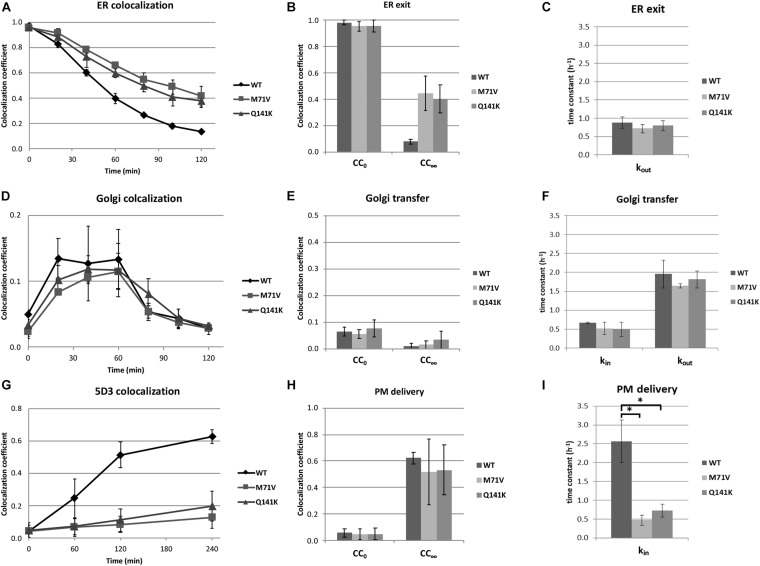
Kinetic analysis of ER to cell surface trafficking of the ABCG2 variants. Colocalization coefficients at various time points were calculated in HeLa cells expressing ABCG2 wt, M71V, or Q141K variants using ER Tracker **(A–C)**, Giantin **(D–F)**, or cell surface labeling with 5D3 **(G–I)** as cellular markers. The kinetic curves shown in Panels **(A,D,G)** were fitted; and the initial values (*CC*_0_), the limits of function (*CC*_∞_) **(B,E,F)**, as well as the time constants **(C,F,I)** were determined. *k*_*in*_ and *k*_*out*_ represent the time constants for entering and exiting the given compartment, respectively. Data represent mean ± SEM of three independent experiments involving 100–120 cells each. Asterisks indicate statistically significant differences (*p* < 0.05).

The kinetics of the various stages of ER to PM trafficking was determined by colocalization analysis using the *CC* as discussed above. To generate data suitable for function fitting, higher time resolution was applied. We found that the release of the polymorphic variants from the ER was restricted as compared to that of the wt (Figure 5A). When the kinetic curves were fitted with an exponential decay, the initial values were alike and close to 1, as expected (Figure 5B). Surprisingly, the time constants did not differ either (Figure 5C), indicating that the rates of ER exit are similar for the wt and the polymorphic variants. In contrast, the limit of the function, the value to which the *CC* values converge, was close to zero in the case of the wt, whereas this figure was about 40% for the M71V and Q141K variants (Figure 5B), suggesting that a substantial fraction of the polymorphic variants is immobile. Colocalization study using the Golgi marker demonstrated that the transit of the polymorphic variants through the Golgi apparatus is similar to that of the wt (Figure 5D). The quantitative analysis of the kinetic curves revealed that neither the initial values, nor the entry and exit rates, nor the convergences differed (Figures 5E,F).

Cell surface labeling of ABCG2 with 5D3 allowed us to determine the kinetics of PM delivery (Figure 5G). Interestingly, the rate of delivery was significantly lower for the polymorphic variants, whereas the initial values and the limits of the function remained unchanged (Figures 5H,I). Unlike using ER or Golgi markers, cell surface labeling with Alexa Four 647 conjugated 5D3 allows us to follow the PM delivery of ABCG2 directly. Accumulation of far-red fluorescence reflects the appearance of ABCG2 on the cell surface. The kinetics of this signal, shown in Supplementary Figure 5, was found to be similar to that seen with the *CC*. Again, the time constants for the cell surface delivery of the polymorphic variants were significantly lower than that for the wt (Supplementary Figure 5C). It can be hypothesized that the half-life of the polymorphic variants in the PM is so short that the antibody is unable to bind to the protein, causing an apparent reduction in the delivery rates. This scenario cannot completely be excluded, but is highly unlikely. Considering that the association constants of the antibodies used for labeling fall into the 0.5–5 × 10^6^ M^–1^ s^–1^ range, labeling of ABCG2 with 1 μg/ml 5D3 is expected to take place within a few minutes, whereas the half-life of the wt ABCG2 in the PM is over 60 h (Peng et al., 2010). Nevertheless, no data on the internalization rates or the PM half-lives of the M71V and Q141K variants are available.

In summary, our data demonstrate that the M71V and Q141K ABCG2 variants have a trafficking deficiency. Specifically, our results suggest that the trafficking of the polymorphic variants is affected at two points, namely, (i) a large fraction of these variants is immobile and retained in the ER, as well as (ii) their PM delivery beyond the Golgi apparatus is delayed. What happens to the ABCG2 variants between the Golgi and the PM remains elusive. One of the possibilities is that a portion of the polymorphic variants gets degraded post-Golgi. It is also conceivable that the variants are retained in an intracellular pool, e.g., in endosomes. Other ABC transporters, such as ABCB11/BSEP and ABCB1/MDR1, have been reported to get delivered to the PM through a large endosomal reservoir (Sai et al., 1999; Kipp and Arias, 2000; Kipp et al., 2001; De Rosa et al., 2004; Wakabayashi et al., 2005; Fu et al., 2007). It is also possible that a fraction of the variants is sequestered intracellularly. Disposal of Q141K-ABCG2 to aggresomes has previously been demonstrated (Basseville et al., 2012). Which one of these processes (or conceivably a combination of these) is responsible for the diminished delivery of the polymorphic ABCG2 variants is yet to be studied. Nonetheless, a fraction of both studied variants unambiguously traffics to the cell surface, although their delivery rate is smaller than that of the wt.

### Effect of Pharmacological Agents on RUSH-ABCG2 Trafficking

**FIGURE 6:**
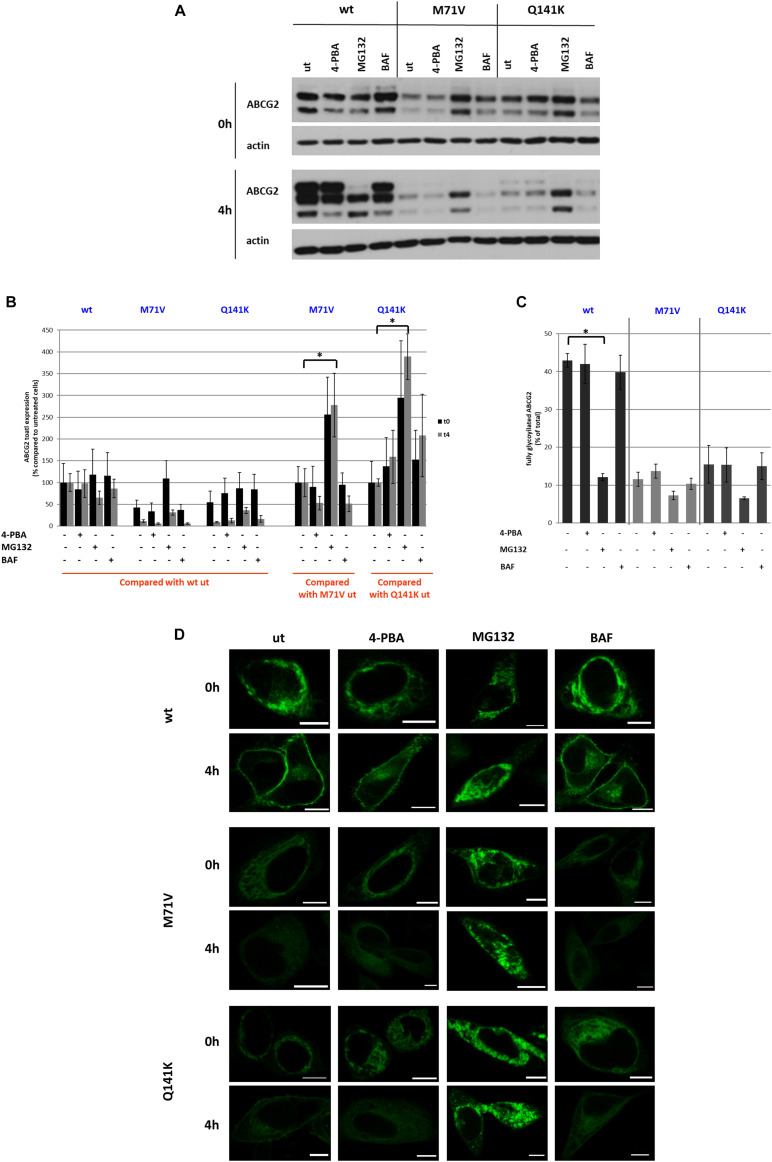
Effect of pharmacological treatments on the RUSH-ABCG2 system. **(A)** Western blot analysis of wt, M71V, and Q141K ABCG2 variants following treatment with 1 mM 4-phenylbutyric acid (4-PBA), 2 μm MG132, or 10 nM Bafilomycin A1 (BAF), compared to the untreated samples (ut). Samples were collected before (0 h) and 4 h after biotin addition (4 h). **(B)** Quantitative analysis of Western blots similar to that shown in Panel **(A)**. The densities were normalized either to the untreated wt sample (0 h), or to the untreated polymorphic variant forms (0 h) as indicated. Data are mean ± SEM of three independent experiments. Asterisks indicate statistically significant differences (*p* < 0.05). **(C)** The fraction of the upper bands were determined by densitometry and expressed as % of total. Data are mean ± SEM. **(D)** Representative confocal microscopy images of HeLa cells expressing the ABCG2 variants pretreated with 4-PBA, MG132, or BAF before (0 h) and 4 h after (4 h) biotin addition. Scale bars represent 10 μm.

To characterize the RUSH-ABCG2 system further, we employed various drugs that influence protein folding and degradation, such as 4-PBA, a chemical corrector, MG132, a proteasome inhibitor, and BAF, a lysosome inhibitor. HeLa cells were pre-treated with these drugs for 24 h following transfection, and then the ER-retained ABCG2 variants were released by biotin. The protein levels in the absence (0 h) and 4 h after the addition of biotin (4 h) were determined by Western blot analysis (Figure 6A). Neither 4-PBA nor BAF affected the levels of any of the three ABCG2 variants significantly (Figures 6A,B). MG132 treatment had no effect on wt ABCG2 expression but increased the protein levels of the polymorphic variants. Normally, when the ABCG2 variants are released from the ER by biotin, an upper band, reflecting the glycosylated form of the protein, appears on the Western blot (see Figures 2E,F). However, following MG132 treatment neither wt ABCG2, nor the polymorphic variants undergo glycosylation (Figures 6A,C). This finding is unexpected, since in previous studies on HeLa cells stably expressing wt and M71V-ABCG2, MG132 treatment resulted in an increase in the general expression of both variants (Zambo et al., 2020). Importantly, the elevation of ABCG2 expression in these cell lines was predominantly due to the increase of the non-glycosylated form, whereas the amount of the glycosylated form remained unaltered. Similar results were obtained with other ABCG2 mutants, such as the F208S and S441N variants when they were treated with MG132 (Nakagawa et al., 2008). Along with these observations, our findings suggest that MG132 blocks ABCG2 routing prior to reaching the Golgi apparatus.

To examine the effect of the abovementioned pharmacological agents on the cellular trafficking of the ABCG2 variants, we imaged both the untreated and the pre-treated cells in the absence or presence of biotin by confocal microscopy. We found that 4-PBA did not substantially alter the trafficking of either the wt or the polymorphic variant ABCG2 constructs within the studied time frame (Figure 6D). Similarly, BAF did not alter their subcellular localization, suggesting that the ABCG2 variants do not undergo lysosomal degradation within 4 h after their release from the ER. Nevertheless, following MG132 treatment, the wt ABCG2, as well as the polymorphic variants were retained intracellularly, even 4 h after biotin addition, as demonstrated by the GFP signal distribution. This explains why MG132 prevented glycosylation of all ABCG2 variants in our previous experiment. If trafficking is blocked and the transporter cannot enter the Golgi apparatus, its glycosylation cannot be completed. Why and how MG132 impedes the release of proteins from the ER remain to be revealed.

Similar to our recent observations using the RUSH constructs, previous studies also demonstrated increased expression levels of the wt and Q141K ABCG2 variants upon MG132 treatment (Furukawa et al., 2009; Basseville et al., 2012), and we also reported the same for M71V-ABCG2 (Zambo et al., 2020). Based on cell surface labeling with 5D3 and confocal microscopy, Furukawa et al. (2009) suggested that inhibition of proteasomal degradation has no effect on the cell surface delivery of the wt ABCG2, while it facilitates the trafficking of the Q141K variant. It should, however, be noted that all of these previous studies employed stable cell lines, which are not suitable for exploring trafficking events, since only the steady-state distribution of the protein can be elucidated in these cells. Transient expression or, even better, synchronous release systems have the potential to reveal the intracellular movements of a protein of interest. To compare stably expressing and transiently transfected cell lines with the cells transfected with RUSH vector in terms of the effect of MG132, we explored the expression and localization of wt ABCG2 in these three cellular models using immunostaining and/or confocal imaging, as well as Western blotting (Supplementary Figure 6). We found that the core/non-glycosylated form of ABCG2 was hardly detectable in the stable cell line, and most of the protein was localized to the PM. Upon MG132 treatment, some extra intracellular staining was observed, and a marked band appeared on the Western blot at the level of the core/non-glycosylated form (middle panels and lanes). The cell surface localization, as well as the intensity of the upper band reflecting the glycosylated form, remained basically unaltered. In contrast, in cells transiently transfected with ABCG2, the level of the glycosylated form markedly decreased following MG132 treatment, and the majority of ABCG2 was localized intracellularly (left panels and lanes). The effect of MG132 in the RUSH system was even more pronounced: no glycosylated band and no PM localized ABCG2 could be observed after the inhibition of proteasomal degradation (right panels and lanes). These experiments indicate that MG132 has no substantial effect on the fraction of ABCG2 that has already reached the PM, but it blocks its release from the ER (or alternatively facilitates its retrograde transport from the Golgi to the ER). Our results also draw attention to the point that steady-state systems, such as cell lines stably expressing the protein of interest, are not suitable for studying dynamic events like protein trafficking, since the distributed protein levels can obscure the observation of transient changes.

## Conclusion

In summary, we have adapted the RUSH system for studying the cellular routing of various ABCG2 variants. This dynamic approach, based on the retention and synchronous release of the protein of interest, allowed us to study the release from the ER, the transfer through the Golgi apparatus and the delivery to the PM separately. In accordance with previous observations, we found that the M71V and Q141K polymorphic variants were expressed at a lower level than wt ABCG2, most likely as a consequence of protein instability and proteasomal degradation. We demonstrated that the trafficking of both the M71V and the Q141K variants are substantially impaired, and the characteristics of their defect are similar. The assay we developed, capable of determining the spatiotemporal distribution of ABCG2 among various cellular compartments, allowed us to identify the particular trafficking steps affected by these polymorphisms. A large portion (about 40%) of the polymorphic variants is immobile, which cannot leave the ER. Also, the delivery of Q141K and M71V to the PM is deferred. Other trafficking parameters, at least those that are involved in the ER to PM routing, remained unaltered. To understand what exactly happens to the polymorphic variants between the Golgi apparatus and the PM requires further investigations. In addition to identifying the specific trafficking defects of various ABCG2 variants, we demonstrated how this system can be used for testing pharmacological agents that potentially influence cellular distributions of these variants. Specifically, the experimental tool we developed in this study is applicable for screening drugs that promote PM delivery of ABCG2 variants with impaired trafficking; thus, it has the potential to support the development of more effective and personalized therapies for gout patients.

## Data Availability Statement

The raw data supporting the conclusions of this article will be made available by the authors, without undue reservation.

## Author Contributions

ZB designed and performed the experiments, analyzed the data, and wrote the manuscript. LH designed the concept of the study and experiments, analyzed the data, and wrote the manuscript. Both authors reviewed the manuscript.

## Conflict of Interest

The authors declare that the research was conducted in the absence of any commercial or financial relationships that could be construed as a potential conflict of interest.

## Supplementary Material

Supplementary Figure 1 Labeling protocols for tracking ABCG2 in various cellular compartments. **(A)** Timeline of ER-Tracker labeling of HeLa cells transfected with RUSH-ABCG2. Red arrow indicates when the cells were subjected to ER-Tracker Red in the absence of biotin. Addition of biotin alone is marked with dark blue arrows, whereas incubation with biotin and ER-Tracker Red is shown in light blue. Yellow flashes mark the time of fixation with 4% paraformaldehyde. **(B)** Timeline of Golgi marker labeling. After fixation, the cells were subjected to immunostaining with an anti-Giantin antibody. **(C)** Timeline of 5D3 labeling. The red arrow here indicates incubation with Alexa Fluor 647 conjugated 5D3 antibody in the presence of Ko143, whereas light blue arrows designate when this mixture was supplemented with biotin. Purple flashes indicate a gentle, 5-min fixation with 1% paraformaldehyde.

Supplementary Figure 2 Maturation of the ABCG2 variants. The RUSH-ABCG2 constructs were expressed in HeLa cells and subjected to biotin. **(A)** Lysates from wt ABCG2-expressing cells without biotin treatment (0 h), as well as 4 and 24 h after biotin addition were subjected to PNGase F digestion. Western blot analysis depicts the comparison between glycosidase-treated and untreated samples. **(B)** Western blots of cell lysates, containing the ABCG2 variants and collected before (0 h), as well as 1, 2, and 4 h after the addition of biotin, were quantitatively analyzed. The fractions of the three bands were determined by densitometry and are expressed as % of total ± SEM (*n* = 3). **(C)** Quantitative analysis of total protein expression of ABCG2 variants before (0 h), as well as 1, 2, and 4 h after biotin addition. Data depict mean ± SEM (*n* = 3).

Supplementary Figure 3 Cell surface labeling of ABCG2 with 5D3 for an extended time. **(A)** Kinetics of cell surface delivery of ABCG2 monitored for 8 h. Experimental conditions are identical to those shown in Figure 3C (also see Supplementary Figure 2C). Briefly, HeLa cells transfected withRUSH-ABCG2-wt were subjected to 5D3 antibody in the presence of Ko143 and biotin (except for time 0, when biotin was omitted). **(B)** Confocal microscopy images of HeLa cells expressing RUSH-ABCG2-wt (green) were labeled with 5D3 antibody (red), and subjected to biotin for 6 and 8 h. An extensive internalization of ABCG2 can be observed. In addition, 5D3 labeling seems to be stable even intracellularly. **(C)** Testing for 5D3 antibody depletion from the labeling medium. Supernatant from cells shown in Panel **(B)** (6 h) were transferred to GFP-ABCG2-expressing HeLa cells. The used supernatant was still able to detect ABCG2 on the cell surface. Scale bars represent 10 μm.

Supplementary Figure 4 Cell surface appearance of the M71V- and Q141K-ABCG2 polymorphic variants. HeLa cells transfected with RUSH-ABCG2-M71V or RUSH-ABCG2-Q141K were cell surface labeled with Alexa Fluor 647 conjugated 5D3 antibody 4 h following biotin addition. The representative confocal fluorescence images of M71V **(A)** and Q141K **(B)** (the same as shown in Figures 4A,B, respectively) were evaluated by intensity profile analysis along the blue lines indicated. The lower panels depict the intensity profiles for GFP (ABCG2–green) and 5D3 labeling (red). Scale bars represent 10 μm.

Supplementary Figure 5 Kinetic analysis of cell surface appearance of the ABCG2 variants using far red fluorescence signal. Integrated far red signals at various time points were determined in the experiments exploring the plasma membrane delivery of the ABCG2. The kinetic curves shown in Panel **(A)** were then fitted with sigmoidal functions. The parameters of the fits, such as the initial values (*F*_0_), the limits of function (*F*_∞_), and the time constants (*k*_*in*_) are presented in Panels **(B,C)**. Data are obtained from three independent experiments involving 100–120 cells each. Error bars represent ± SEM. Asterisks indicate statistically significant differences (*p* < 0.05).

Supplementary Figure 6 Effect of the proteasome inhibitor MG132 on the localization and glycosylation of ABCG2 in stable and transient cell lines. HeLa cells transiently transfected with ABCG2-wt (Orban et al., 2008) or RUSH-ABCG2-wt, and HeLa cells stably expressing ABCG2 (Zambo et al., 2020) were subjected to 2 μM MG132 overnight. **(A)** Representative confocal microscopy images depict untreated (upper panels) and MG132 treated cells (lower panels) of the various cellular models. Scale bars represent 20 μm. **(B)** Western blot analysis of lysates of untreated and MG132 treated cells. Each sample contains 50 μg protein. The molecular weight of mature ABCG2 is 72 kDa, while that of its GFP-tagged counterpart is about100 kDa.

## Abbreviations

4-PBA: 4-phenylbutyric acid
ABCG2: ATP-binding transporter subfamily G member 2
BAF: Bafilomycin A1
BCRP: breast cancer resistance protein
CC: colocalization coefficient
CFTR: cystic fibrosis transmembrane conductance regulator
CMV: cytomegalovirus
DMSO: dimethyl sulfoxide
Endo H: endoglycosidase H
ER: endoplasmic reticulum
GFP: green fluorescent protein
Ii: invariant chain
IRES: internal ribosomal entry site
IVS: intervening sequence
MHC: major histocompatibility complex
PM: plasma membrane
PNGase F: N-glycosidase F
RUSH: retention using selective hooks
SBP: streptavidin-binding peptide
Str: streptavidin
wt: wild type.
