# Supplementary figures and images for: Identification of Specific Trafficking Defects of Naturally Occurring Variants of the Human ABCG2 Transporter
Source: Front Cell Dev Biol. 2021 Feb 9;9:615729. doi: 10.3389/fcell.2021.615729 (PMC7900420; doi:10.3389/fcell.2021.615729)

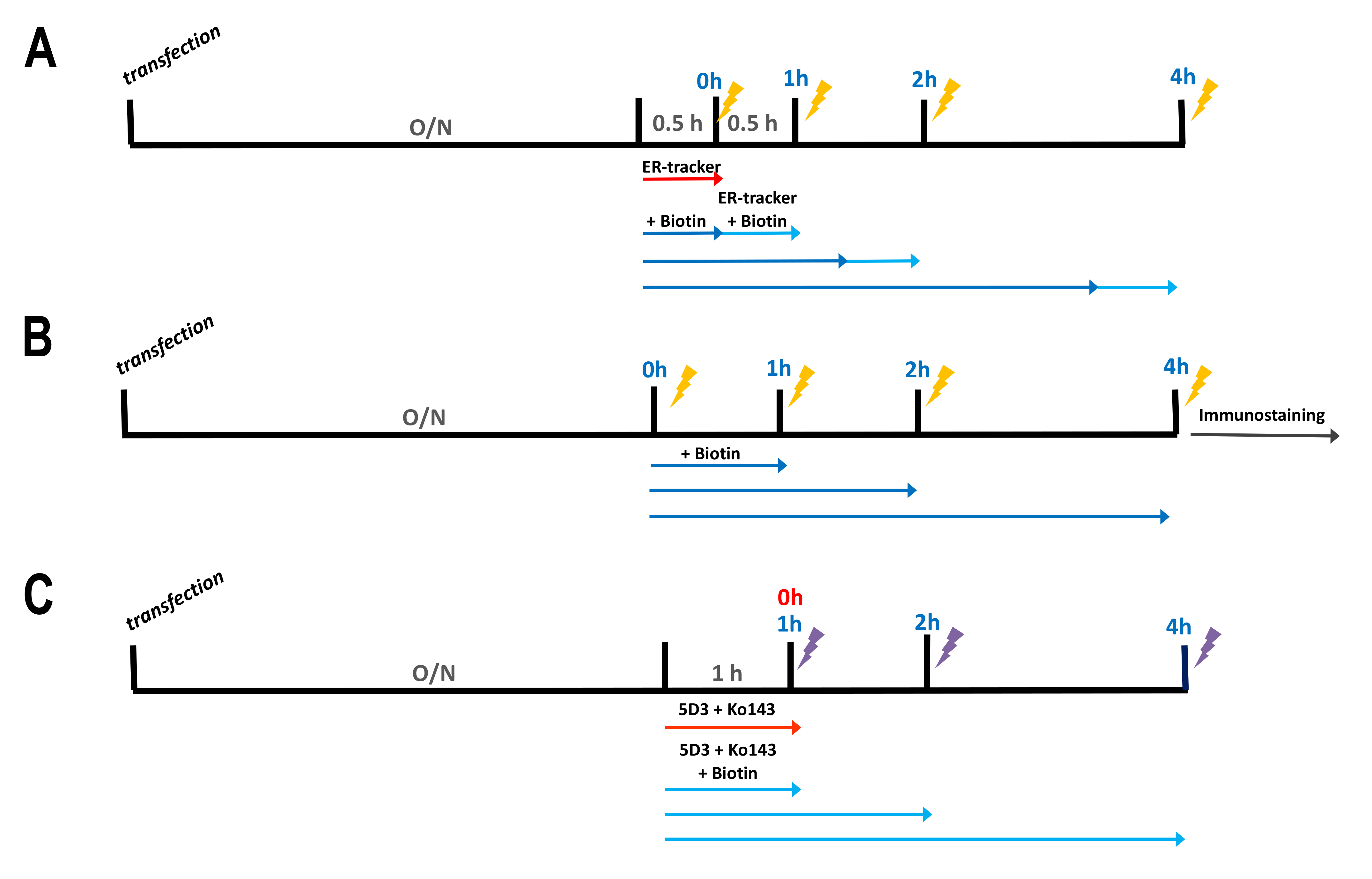

Supplement: Supplementary Figure 1 — Labeling protocols for tracking ABCG2 in various cellular compartments. (A) Timeline of ER-Tracker labeling of HeLa cells transfected with RUSH-ABCG2. Red arrow indicates when the cells were subjected to ER-Tracker Red in the absence of biotin. Addition of biotin alone is marked with dark blue arrows, whereas incubation with biotin and ER-Tracker Red is shown in light blue. Yellow flashes mark the time of fixation with 4% paraformaldehyde. (B) Timeline of Golgi marker labeling. After fixation, the cells were subjected to immunostaining with an anti-Giantin antibody. (C) Timeline of 5D3 labeling. The red arrow here indicates incubation with Alexa Fluor 647 conjugated 5D3 antibody in the presence of Ko143, whereas light blue arrows designate when this mixture was supplemented with biotin. Purple flashes indicate a gentle, 5-min fixation with 1% paraformaldehyde. [file Image_1.tif]

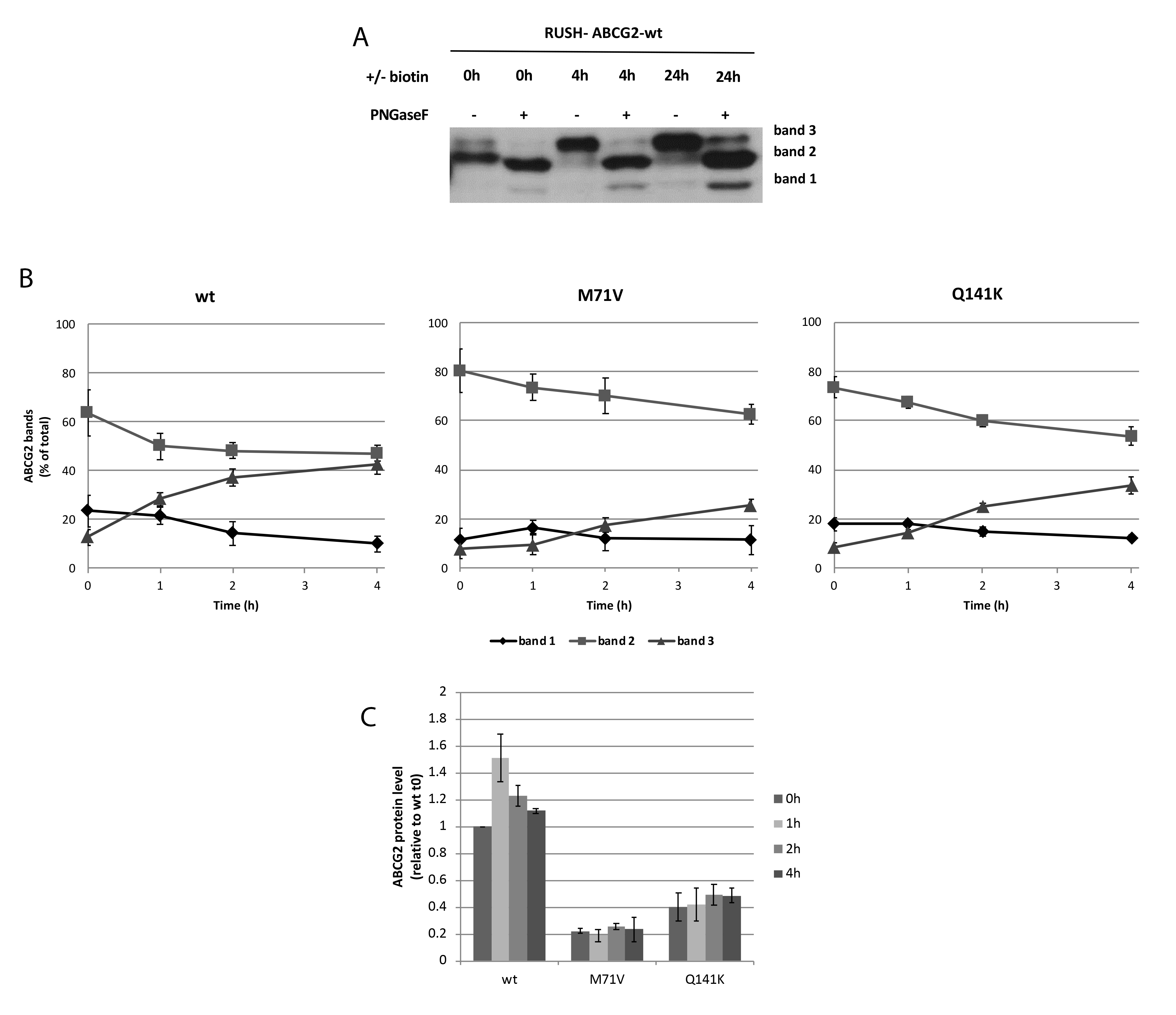

Supplement: Supplementary Figure 2 — Maturation of the ABCG2 variants. The RUSH-ABCG2 constructs were expressed in HeLa cells and subjected to biotin. (A) Lysates from wt ABCG2-expressing cells without biotin treatment (0 h), as well as 4 and 24 h after biotin addition were subjected to PNGase F digestion. Western blot analysis depicts the comparison between glycosidase-treated and untreated samples. (B) Western blots of cell lysates, containing the ABCG2 variants and collected before (0 h), as well as 1, 2, and 4 h after the addition of biotin, were quantitatively analyzed. The fractions of the three bands were determined by densitometry and are expressed as % of total ± SEM (n = 3). (C) Quantitative analysis of total protein expression of ABCG2 variants before (0 h), as well as 1, 2, and 4 h after biotin addition. Data depict mean ± SEM (n = 3). [file Image_2.tif]

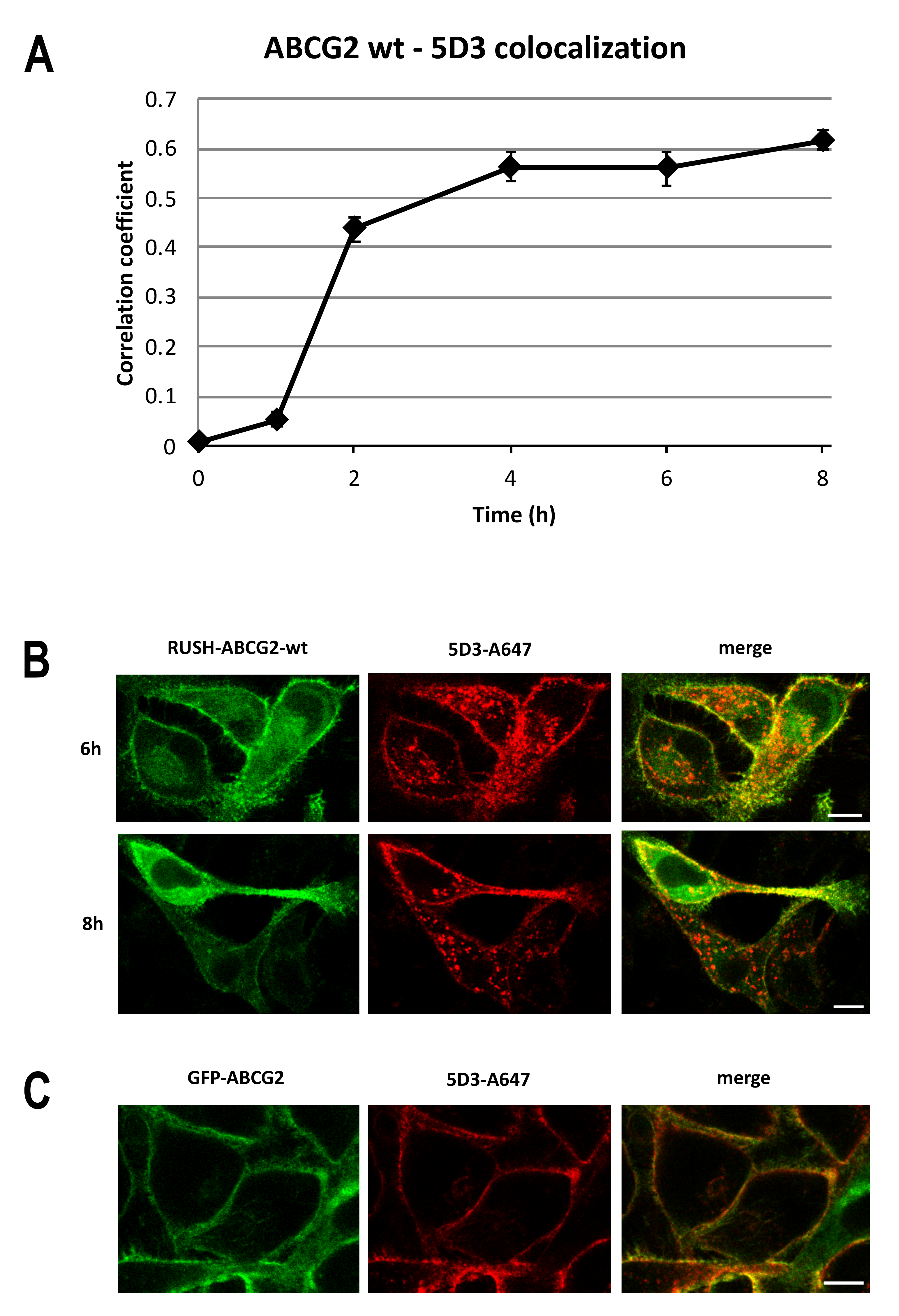

Supplement: Supplementary Figure 3 — Cell surface labeling of ABCG2 with 5D3 for an extended time. (A) Kinetics of cell surface delivery of ABCG2 monitored for 8 h. Experimental conditions are identical to those shown in Figure 3C (also see Supplementary Figure 2C). Briefly, HeLa cells transfected withRUSH-ABCG2-wt were subjected to 5D3 antibody in the presence of Ko143 and biotin (except for time 0, when biotin was omitted). (B) Confocal microscopy images of HeLa cells expressing RUSH-ABCG2-wt (green) were labeled with 5D3 antibody (red), and subjected to biotin for 6 and 8 h. An extensive internalization of ABCG2 can be observed. In addition, 5D3 labeling seems to be stable even intracellularly. (C) Testing for 5D3 antibody depletion from the labeling medium. Supernatant from cells shown in Panel (B) (6 h) were transferred to GFP-ABCG2-expressing HeLa cells. The used supernatant was still able to detect ABCG2 on the cell surface. Scale bars represent 10 μm. [file Image_3.tif]

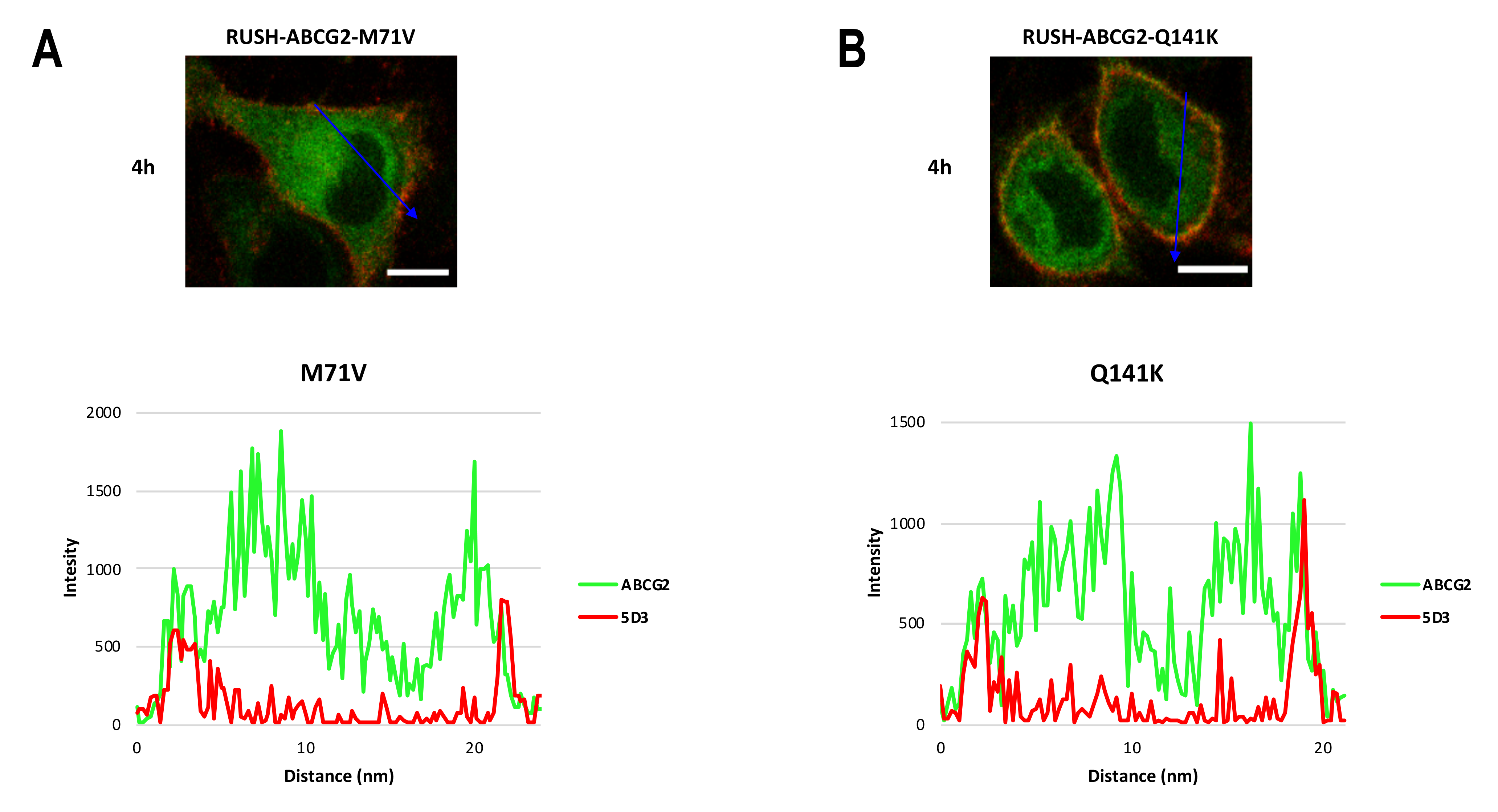

Supplement: Supplementary Figure 4 — Cell surface appearance of the M71V- and Q141K-ABCG2 polymorphic variants. HeLa cells transfected with RUSH-ABCG2-M71V or RUSH-ABCG2-Q141K were cell surface labeled with Alexa Fluor 647 conjugated 5D3 antibody 4 h following biotin addition. The representative confocal fluorescence images of M71V (A) and Q141K (B) (the same as shown in Figures 4A,B, respectively) were evaluated by intensity profile analysis along the blue lines indicated. The lower panels depict the intensity profiles for GFP (ABCG2–green) and 5D3 labeling (red). Scale bars represent 10 μm. [file Image_4.tif]

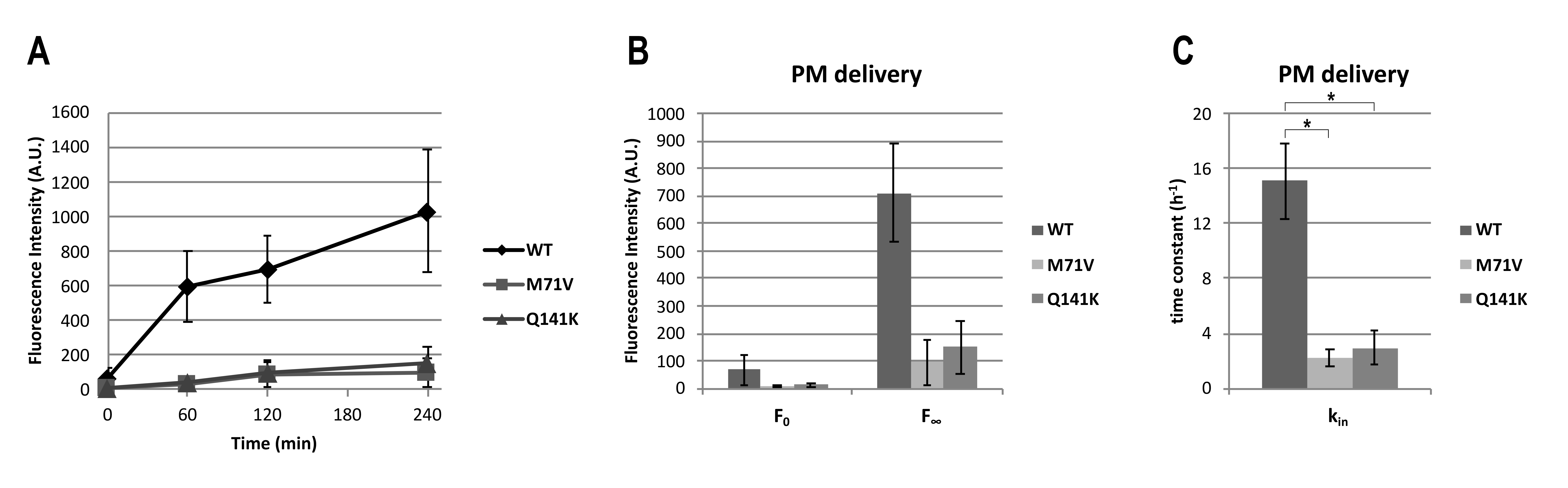

Supplement: Supplementary Figure 5 — Kinetic analysis of cell surface appearance of the ABCG2 variants using far red fluorescence signal. Integrated far red signals at various time points were determined in the experiments exploring the plasma membrane delivery of the ABCG2. The kinetic curves shown in Panel (A) were then fitted with sigmoidal functions. The parameters of the fits, such as the initial values (F0), the limits of function (F∞), and the time constants (kin) are presented in Panels (B,C). Data are obtained from three independent experiments involving 100–120 cells each. Error bars represent ± SEM. Asterisks indicate statistically significant differences (p < 0.05). [file Image_5.tif]

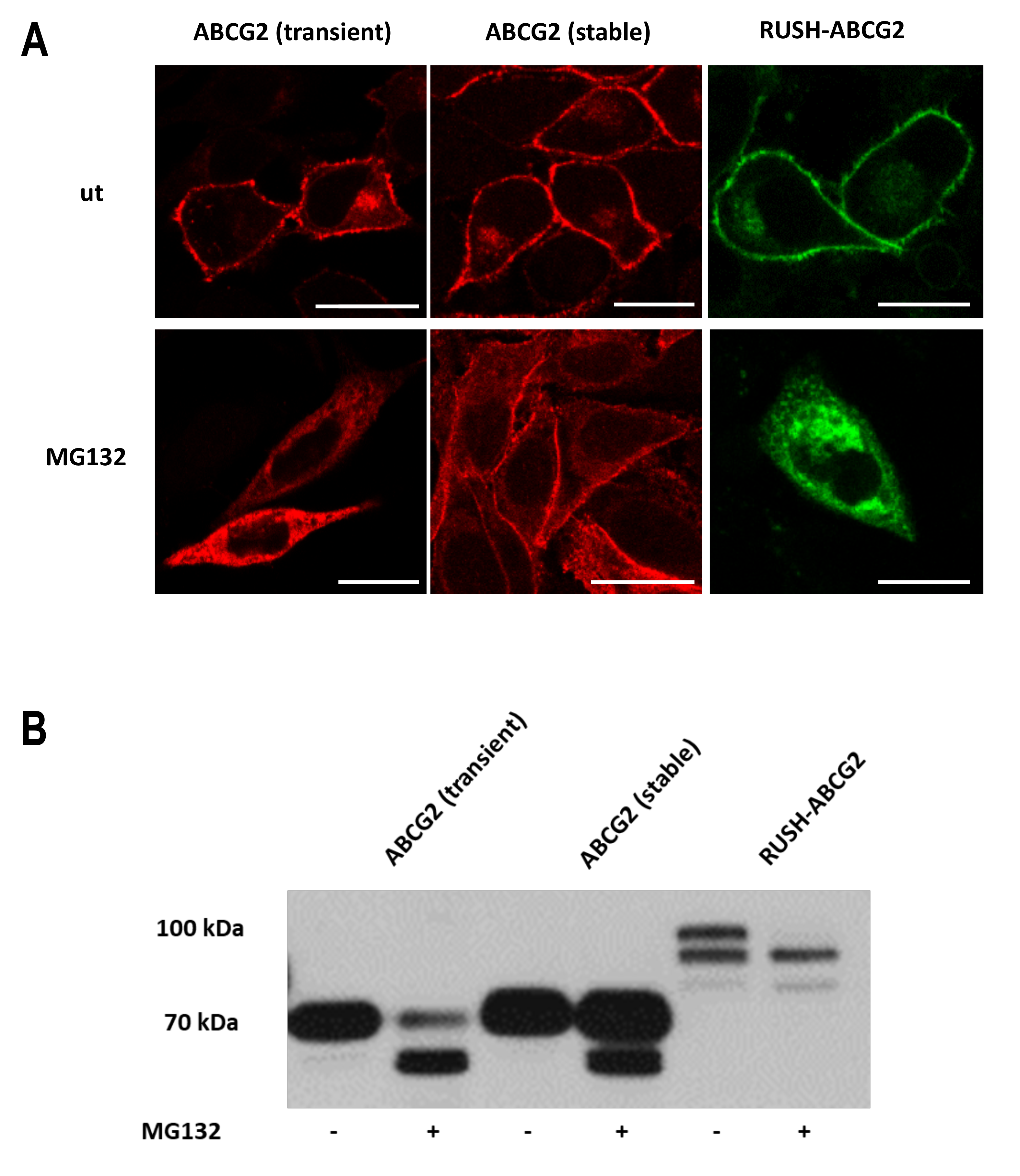

Supplement: Supplementary Figure 6 — Effect of the proteasome inhibitor MG132 on the localization and glycosylation of ABCG2 in stable and transient cell lines. HeLa cells transiently transfected with ABCG2-wt (Orban et al., 2008) or RUSH-ABCG2-wt, and HeLa cells stably expressing ABCG2 (Zambo et al., 2020) were subjected to 2 μM MG132 overnight. (A) Representative confocal microscopy images depict untreated (upper panels) and MG132 treated cells (lower panels) of the various cellular models. Scale bars represent 20 μm. (B) Western blot analysis of lysates of untreated and MG132 treated cells. Each sample contains 50 μg protein. The molecular weight of mature ABCG2 is 72 kDa, while that of its GFP-tagged counterpart is about100 kDa. [file Image_6.tif]
